# A Proteomics Analysis of Calmodulin-Binding Proteins in *Dictyostelium discoideum* during the Transition from Unicellular Growth to Multicellular Development

**DOI:** 10.3390/ijms22041722

**Published:** 2021-02-09

**Authors:** William D. Kim, Shyong Q. Yap, Robert J. Huber

**Affiliations:** 1 Environmental and Life Sciences Graduate Program, Trent University, Peterborough, ON K9L 0G2, Canada; williamkim@trentu.ca (W.D.K.); shyongyap@trentu.ca (S.Q.Y.); 2 Department of Biology, Trent University, Peterborough, ON K9L 0G2, Canada

**Keywords:** calmodulin, calmodulin-binding proteins, *Dictyostelium discoideum*, immunoprecipitation, mass spectrometry

## Abstract

Calmodulin (CaM) is an essential calcium-binding protein within eukaryotes. CaM binds to calmodulin-binding proteins (CaMBPs) and influences a variety of cellular and developmental processes. In this study, we used immunoprecipitation coupled with mass spectrometry (LC-MS/MS) to reveal over 500 putative CaM interactors in the model organism *Dictyostelium discoideum*. Our analysis revealed several known CaMBPs in *Dictyostelium* and mammalian cells (e.g., myosin, calcineurin), as well as many novel interactors (e.g., cathepsin D). Gene ontology (GO) term enrichment and Search Tool for the Retrieval of Interacting proteins (STRING) analyses linked the CaM interactors to several cellular and developmental processes in *Dictyostelium* including cytokinesis, gene expression, endocytosis, and metabolism. The primary localizations of the CaM interactors include the nucleus, ribosomes, vesicles, mitochondria, cytoskeleton, and extracellular space. These findings are not only consistent with previous work on CaM and CaMBPs in *Dictyostelium*, but they also provide new insight on their diverse cellular and developmental roles in this model organism. In total, this study provides the first in vivo catalogue of putative CaM interactors in *Dictyostelium* and sheds additional light on the essential roles of CaM and CaMBPs in eukaryotes.

## 1. Introduction

Calcium is an essential ion within eukaryotic cells that is housed in all organelles but is most prominently found in the endoplasmic reticulum [1,2]. The mechanistic capabilities of calcium are mediated by its binding to the EF-hand regions of calcium-binding proteins [3]. Calmodulin (CaM) is an essential calcium-binding protein that is conserved across eukaryotes [4]. CaM binds to CaM-binding proteins (CaMBPs) in a calcium-dependent or calcium-independent manner via calmodulin-binding domains (CaMBDs) that include hydrophobic amino acid calcium-dependent motifs or calcium-independent IQ motifs [5,6].

The functions of CaMBPs are diverse and include, but are not limited to, kinases/phosphatases, heat-shock proteins, and cytoskeletal proteins [7,8]. As a result, CaM and CaMBPs are associated with several essential processes in eukaryotic cells such as autophagy, apoptosis, cell cycle progression, cell proliferation, cell differentiation, and cytoskeletal organization [8]. Not surprisingly, CaM and CaMBPs have been linked to several human diseases including Alzheimer’s disease, heart disease, and the neuronal ceroid lipofuscinoses (NCLs, commonly known as Batten disease) [9,10,11].

CaM-dependent events have been well studied in a variety of organisms. One such organism is the social amoeba *Dictyostelium discoideum* [6,12]. *Dictyostelium* has a 24-h life cycle that consists of unicellular and multicellular phases [13]. In the unicellular growth phase, *Dictyostelium* cells are in a nutrient-rich condition and undergo cellular division via mitosis. When nutrients are scarce or depleted, *Dictyostelium* cells centralize into a single mound via cyclic adenosine monophosphate (cAMP) chemoattractant signalling. Through a series of multicellular structural changes, a fruiting body is formed that is comprised of a droplet of spores that is held atop a slender stalk. When introduced into an environment containing nutrients, the spores germinate, and the life cycle restarts. *Dictyostelium* is an exceptional model system for studying conserved cellular and developmental processes as well as the functions of proteins associated with human diseases [13,14]. In *Dictyostelium*, CaM (also known as CalA), and its associated CaMBPs are linked to a variety of cellular and developmental processes [6,12,15].

Historically, CaMBPs in *Dictyostelium* have been revealed through directed studies aimed at confirming whether a suspected protein binds CaM [16,17,18,19,20,21,22,23,24,25,26]. In addition, the CaM-binding overlay technique (CaMBOT), which involves separating proteins by SDS-PAGE and then performing a gel overlay with recombinant radiolabelled CaM (35[S]-CaM), has been useful for identifying putative CaMBPs in a biological sample [27]. While these approaches have been useful for confirming putative CaMBPs and revealing novel interactors (e.g., CaMBOT), a global in vivo analysis of CaM interactors has not previously been performed in *Dictyostelium*. Here, we used immunoprecipitation (IP) coupled with liquid chromatography-mass spectrometry/mass spectrometry (LC-MS/MS) to reveal over 500 putative CaM interactors in growth-phase cells and cells starved for 6 h (representing the early stages of *Dictyostelium* development). The proteins we identified may bind CaM directly or may interact with CaMBPs that were pulled down with CaM in the IP. Our study not only confirms CaMBPs previously identified through in vitro methods, but it also identifies novel interactors that extend our understanding of CaM and CaMBP function in *Dictyostelium*.

## 2. Results

### 2.1. Immunoprecipitation of CaM from Growth-Phase and Starved Dictyostelium Cells

**Figure 1:**
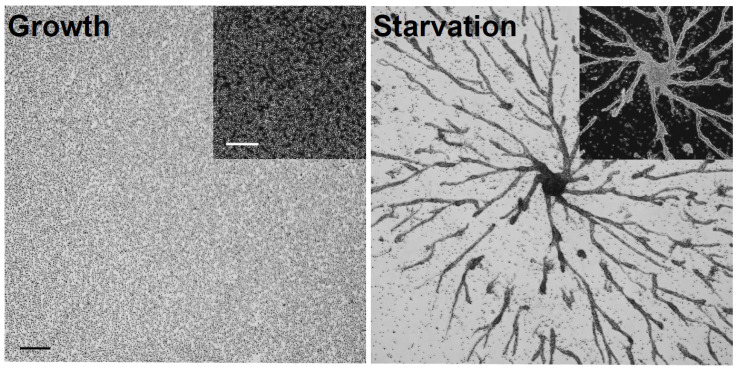
Growth and starvation of *Dictyostelium* cells. Cells were grown overnight in Petri dishes to confluency, after which time, growth-phase cells and cells starved for 6 h in KK2 buffer were harvested. The images shown are representative of three biological replicates. Scale bar = 250 µm.

**Figure 2:**
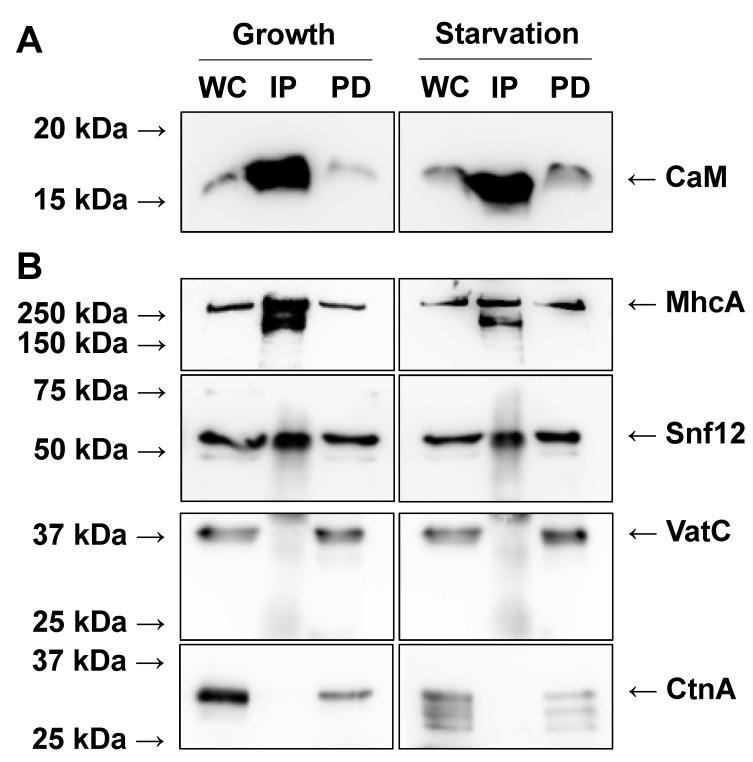
Validation of mass spectrometry results by western blotting. Calmodulin (CaM) immunoprecipitation (IP) fractions from growth-phase and starved cells were separated by SDS-PAGE and analyzed by western blotting. (**A**) Western blots probed with anti-CaM. (**B**) Western blots probed with anti-MhcA (myosin II heavy chain, positive control), anti-Snf12 (SWI/SNF protein 12, positive control), anti-VatC (catalytic subunit of V-ATPase, negative control), and anti-CtnA (countin, negative control). Molecular weight markers (in kDa) are shown to the left of each blot. WC, whole cell lysate (7 µg total protein). IP, CaM IP fraction (11 µL). PD, protein-depleted fraction (7 µg total protein).

To determine putative CaM interactors during growth and the early stages of *Dictyostelium* development, we deposited cells in Petri dishes and submerged them in HL5 growth medium overnight at room temperature. The following day, confluent growth-phase cells were harvested (Figure 1). Cells were also starved for 6 h in KK2 buffer and harvested (Figure 1). After 6 h, cells were aggregating into multicellular mounds, which was consistent with the normal timing of *Dictyostelium* development [28]. A total of three biological replicates were harvested for both growth and starved conditions. CaM was immunoprecipitated from each biological replicate using a well-established antibody that was previously generated against *Dictyostelium* CaM [29]. Western blotting confirmed the immunoprecipitation of CaM from growth-phase and starved cells (Figure 2A).

### 2.2. Mass Spectrometry Reveals CaM Interactors during Growth and Starvation

CaM IP samples from growth-phase and starved cells were analyzed by mass spectrometry (LC-MS/MS), which revealed 517 putative CaM interactors during growth and 521 putative interactors during starvation (Supplementary Materials Table S1). 374 CaM interactors were common to both conditions, with 143 proteins identified only in growth samples, and 147 proteins unique to starved samples (Table S2). Mass spectrometry detected CaM in all biological replicates that were analyzed (Table S1). The *Dictyostelium* genome also encodes a calcium-binding CaM-like protein called CalB, that has about 50% similarity to other CaMs [30]. Mass spectrometry did not detect CalB in CaM IP fractions. To validate the mass spectrometry results, we confirmed that proteins present in our dataset could be detected via western blotting (positive controls). Conversely, we also validated that proteins absent in our dataset could not be detected (negative controls). For our positive controls, we confirmed the presence of myosin II heavy chain (MhcA) and SWI/SNF protein 12 (Snf12) in CaM IP fractions from growth and starved samples (Figure 2B). MhcA is a known CaMBP in *Dictyostelium* [31]. Previous work showed that Snf12, which is a homolog of yeast SNF12 and human SMARCD1 (involved in chromatin remodelling), localizes to the nucleolus and is a potential binding partner of nucleomorphin A (NumA), a previously identified CaMBP [18,32]. For our negative controls, we confirmed the absence of the catalytic subunit of V-ATPase (VatC) and countin (CtnA) in CaM IP samples since neither of these proteins were revealed in our mass spectrometry analysis or are predicted to bind to CaM (Figure 2B). In total, these results support the specificity of the CaM IP and the catalogue of putative interactors revealed by mass spectrometry (i.e., the presence or absence of specific proteins from the dataset were validated by western blotting).

### 2.3. Mass Spectrometry Reveals Previously Known CaMBPs in Dictyostelium

Several of the proteins identified by mass spectrometry have the gene ontology (GO) term identifier “calmodulin-binding”. These include known or predicted CaMBPs in *Dictyostelium* including calcineurin (CanA), WW domain-containing protein A (DwwA), histone H1 (H1), proteins in the myosin heavy chain family (MhcA, MyoA, MyoE, MyoJ), Ras GTPase-activating-like protein (RgaA), and 60S ribosomal protein L19 (Rpl19) (Table 1, Table S1) [16,17,21,31,33]. All these proteins were detected in growth-phase and starved cells except CanA and RgaA, which were present only in starved samples (Table 1, Table S1). The online bioinformatics resource for the *Dictyostelium* research community, dictyBase, provides accurate and updated annotations for curated genes and proteins [34]. Three proteins with the descriptor “calmodulin” or “calmodulin-binding” were revealed by mass spectrometry and included H1 (discussed above), guanine exchange factor for rac II (GxcII), and guanine exchange factor for rac JJ (GxcJJ) (Table 1, Table S1). All three proteins were present in growth and starved samples except GxcJJ, which was only detected in CaM IP fractions isolated from starved cells (Table 1, Table S1). In addition to the proteins described above, several known or predicted CaMBPs reviewed by O’Day et al [6] were detected by mass spectrometry including myosin heavy chain kinase B (MhkB) (starved only), the V-ATPase membrane subunit (VatM) (growth only), and additional members of the myosin heavy chain family (MyoB, MyoC, MyoD, MyoI) (Table 2, Table S1). MyoB, MyoD, and MyoI were detected in CaM IP fractions from both growth-phase and starved cells, while MyoC was detected only in growth samples (Table 2, Table S1). Finally, mass spectrometry also revealed homologs of known CaMBPs in mammals such as casein kinase I and II (Cak1-1, Cak1-2, CasK, Csnk2b), which were identified in both growth and starved samples [35]. Together, these findings show that mass spectrometry identified established CaMBPs in *Dictyostelium*, which provides additional evidence supporting the list of CaM interactors.

### 2.4. Bioinformatic Analyses Highlight the Diverse Cellular Roles of CaM Interactors in Dictyostelium

To further analyze our list of putative CaM interactors (Table S1), we performed a GO term enrichment analysis (Tables S3 and S4). When assessing “biological process”, 65% (337/517) of proteins in growth samples and 61% (318/517) of proteins in starved samples are associated with metabolic processes (Table 3). Other highly enriched GO term categories included gene expression (45% growth, 44% starved), biosynthetic process (42% growth, 39% starved), translation (28% growth, 27% starved), cytoskeleton organization (10% growth, 12% starved), endocytosis (6% growth, 6% starved), and cytokinesis (3% growth, 4% starved) (Table 3). 

We also examined the “molecular function” of the CaM interactors. Most of the proteins from both growth and starved samples are involved in binding (67% growth, 68% starved) (e.g., nucleic acid, ion, RNA, anion, carbohydrate-derivative, ATP) (Table 4). In growth samples, 39% (202/517) of the proteins have catalytic activity (Table 4). Interestingly, this GO term category was not enriched in starved samples. However, CaM interactors in both growth and starved samples have hydrolase activity (14% growth, 12% starved), pyrophosphatase activity (14% growth, 12% starved), and ATPase activity (7% growth, 7% starved) (Table 4). Like catalytic activity, GTP binding and GTPase activity were only enriched for proteins identified in growth samples (Table 4). In contrast, calcium-binding activity was only enriched in starved samples (Table 4). 

The localizations of proteins identified in growth and starved samples were diverse. The largest proportion of proteins in both samples localize to the nucleus (28% growth, 30% starved) (Table 5). The next highly enriched GO term category was cytosol, which includes the fluid enclosed within the plasma membrane (24% growth, 23% starved) (Table 5). Other highly enriched GO term categories included ribosome (24% growth, 22% starved), vesicle (20% growth, 20% starved), mitochondrion (17% growth, 12% starved), extracellular region (13% growth, 13% starved), and cytoskeleton (9% growth, 10% starved) (Table 5). In addition, some GO terms were only associated with either growth or starved samples. For example, mitochondrial inner membrane and mitochondrial respiratory chain were only enriched in growth samples (Table 5). In contrast, cell periphery was only enriched for proteins in starved samples (Table 5). 

**Figure 3:**
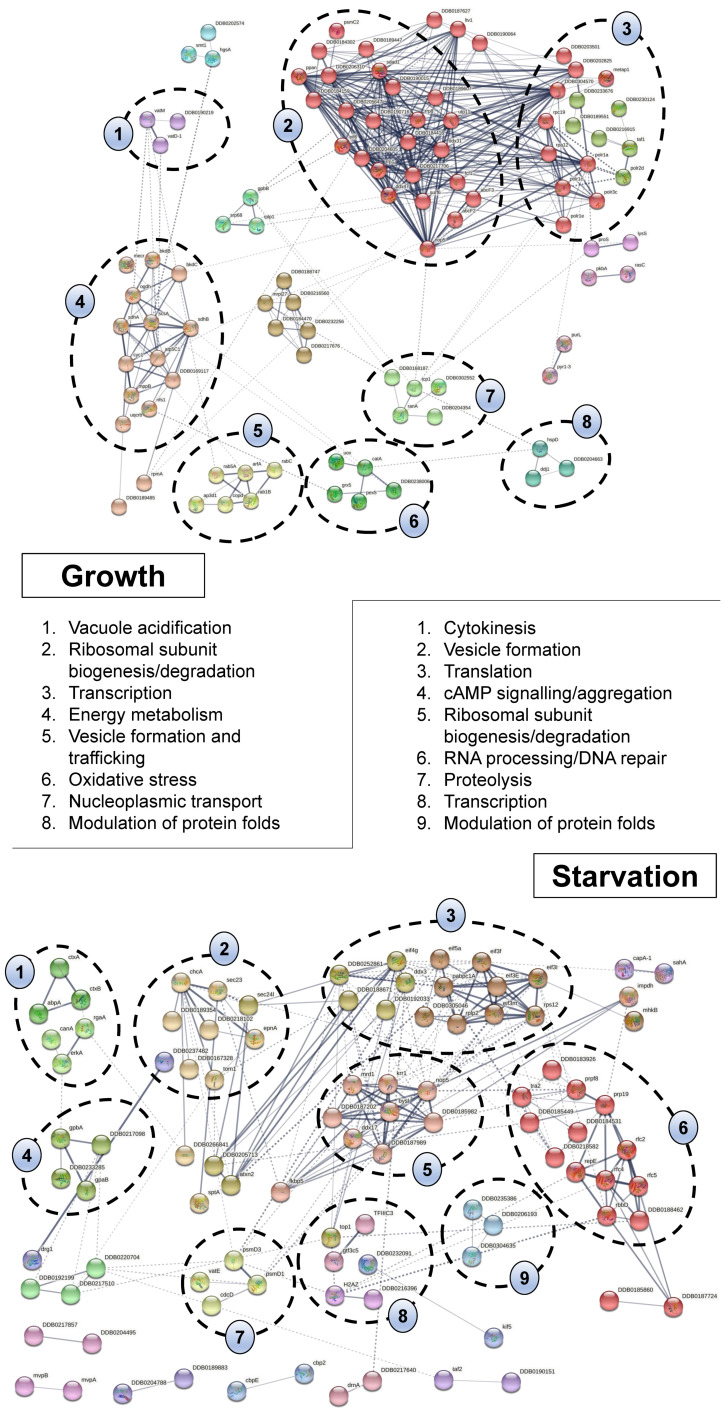
Search Tool for the Retrieval of Interacting proteins (STRING) analysis of putative calmodulin (CaM) interactors unique to growth and starvation. The lines between protein nodes represent interactions under a threshold of medium interacting confidence score (0.400). The edges represent the predicted functional associations. Clustering was performed with an MCL inflation parameter value of 2 with some protein nodes moved to other clusters due to the protein description matching the identified pathway. Clustered proteins are shown in numbered groups.

To gain additional insight into the pathways that the putative CaM interactors belong to, the proteins unique to each condition (Table S2) were analyzed using the Search Tool for the Retrieval of Interacting proteins (STRING) database [36]. STRING predicts protein associations based on direct interactions or participation in common biological pathways. STRING analysis on the list of CaM interactors unique to growth revealed eight processes including vacuolar acidification, energy metabolism, vesicle formation and trafficking, oxidative stress, nucleoplasmic transport, protein-fold regulation, transcription, and ribosomal subunit synthesis and degradation (Figure 3). Nine processes were identified for proteins unique to starvation including cytokinesis, cAMP signalling, vesicle formation, proteolysis, translation, ribosomal subunit synthesis and degradation, transcription, modulation of protein folds, and RNA processing/DNA repair responses (Figure 3). In total, these results highlight the diverse localizations and functions of the putative CaM interactors in *Dictyostelium*.

### 2.5. Mass Spectrometry Reveals Novel CaM Interactors in Dictyostelium with Biomedical Signficance

Mass spectrometry revealed novel CaM interactors with biomedical significance. One example was the *Dictyostelium* homolog of human cathepsin D (human: CTSD; *Dictyostelium*: CtsD), which functions as a lysosomal aspartyl protease, and was present in both growth and starved samples. Mutations in *CTSD* are associated with several human diseases including breast cancer, Alzheimer’s disease, Parkinson’s disease, and a subtype of NCL (Batten disease) [37,38,39,40]. In addition, the *Dictyostelium* homolog human hexosaminidase A (HEXA), N-acetylglucosaminidase A (NagA), was detected in growth samples. Mutations in *HEXA* cause Tay-Sachs disease in humans, which is a neurological disorder characterized by an accumulation of GM2 gangliosides in neurons [41]. Combined, these findings provide additional evidence supporting the biomedical significance of CaM and CaMBPs. 

## 3. Discussion

In this study, we used a highly specific and well-characterized antibody to immunoprecipitate CaM (also known as CalA) from growth-phase and starved *Dictyostelium* cells. We then used mass spectrometry (LC-MS/MS) to reveal over 500 putative CaM interactors in IP fractions. CaM was detected in all IP fractions that were analyzed by mass spectrometry. CalB, a CaM-like protein that shares about 50% similarity with other CaMs was not, indicating that the anti-CaM antibody was specific for CalA. We used western blotting to validate the accuracy of the mass spectrometry analysis. Specifically, we confirmed the presence of MhcA and Snf12 in CaM IP fractions and the absence of VatC and CtnA. The list of proteins was further validated by the presence of known CaMBPs in the dataset including CanA, H1, and proteins belonging to the myosin family, among others. In addition, our analysis revealed several known CaMBPs as well as homologs of proteins associated with human diseases. Finally, bioinformatic analyses highlighted key processes mediated by the putative CaM interactors, thus enhancing our understanding of CaM-mediated signal transduction in *Dictyostelium*.

In *Dictyostelium*, CaM and CaMBPs have been linked to a variety of cellular and developmental processes including mitosis, phagocytosis, autophagy, osmoregulation, cell motility and chemotaxis, cell differentiation, and spore germination [12]. Here, GO term enrichment and STRING analyses linked the putative CaM interactors to a diversity of cellular processes including, but not limited to, metabolism, gene expression, biosynthetic processes, cytoskeleton organization, endocytosis, oxidative stress, and cytokinesis. Many ribosomal proteins were also detected in growth and starved samples, which is consistent with similar large-scale studies on putative CaMBPs in human cells [7]. GO term enrichment analyses also revealed that the putative CaM interactors localize throughout the cell, especially in nuclei, ribosomes, vesicles, mitochondria, and the cytoskeleton. In addition, 13% of the putative CaM interactors localize outside the cell. Consistent with this finding, extracellular CaM has been reported in *Dictyostelium* where it regulates growth and cAMP-mediated chemotaxis [24,42]. STRING analysis of proteins unique to starvation revealed 6 proteins linked to cytokinesis, which aligns with previous findings showing that a round of cytokinesis occurs early in development to allow multinucleated cells to complete their final growth-stage cell cycle [43,44]. Combined, these findings are consistent with the previously reported localizations of CaMBPs in *Dictyostelium* and the cellular and developmental roles of CaM and CaMBPs in *Dictyostelium* [6,12]. 

The functions of CaM and CaMBPs in mitosis and cytokinesis in *Dictyostelium* have been well documented [12,45]. These roles are consistent with the nuclear localization of CaM and CaMBPs such as Snf12 and Cbp4a, which were detected in CaM IP fractions [26,32,46]. In this study, mass spectrometry revealed DwwA as a CaM interactor in both growth and starved cells. DwwA contains domains that act as scaffolds for proteins involved in cytokinesis [21]. Previous work also demonstrated the importance of CaM-dependent phosphorylation and CaMBPs in regulating folic acid and cAMP-mediated chemotaxis in *Dictyostelium* [47]. Not surprisingly, proteins with catalytic or hydrolase activity were identified by mass spectrometry including phospholipase C, phosphatidylinositol-3-phosphatase, 3-hydroxyisobutyryl-CoA hydrolase, alpha mannosidase, and Ras proteins, among others. Cytoskeletal proteins (e.g., members of the myosin family), which are essential for cell motility and chemotaxis, were also identified by mass spectrometry. For example, annexin A7 was identified as a putative CaM interactor in starved cells. In mice, annexin A7 has been linked to the inflammatory response and calcium homeostasis in the brain [48,49]. In humans, annexin A7 appears to have a tumour-suppressive role in some forms of cancer (e.g., glioblastoma), but in others, it seems to promote malignancy (e.g., gastric cancer) [50]. Recently, CaM and annexin were shown to regulate wound repair in *Dictyostelium* by facilitating the accumulation of actin at the wound site [51]. In total, these observations highlight the essential roles of CaMBPs in processes regulated by the cytoskeleton.

The *Dictyostelium* homologs of human HEXA (NagA) and CTSD (CtsD) were identified in CaM IP fractions. Mutations in *HEXA* cause Tay-Sachs disease, while mutations in *CTSD* are associated with breast cancer, Alzheimer’s disease, Parkinson’s disease, and a subtype of NCL called CLN10 disease [37,38,39,40,41]. The identification of NagA and CtsD in *Dictyostelium* CaM IP fractions suggests that CaM-mediated signalling may play an important role in the pathology underlying both diseases. Consistent with these findings, previous work identified two regions in human CTSD that contain putative CaMBDs [11]. In addition, CTSD function has been linked to calcineurin, a known CaMBP in humans [52]. CTSD, together with calcineurin, have been shown to protect against alpha-synuclein toxicity in a yeast model of Parkinson’s disease [53]. Calcineurin has also been linked to autoimmune diseases (e.g., rheumatoid arthritis), psychiatric diseases (e.g., schizophrenia, bipolar disorder), and hypertension [54,55,56]. Importantly, the *Dictyostelium* homolog of human calcineuin, CanA, was identified by mass spectrometry in starved cells. Mass spectrometry also identified the *Dictyostelium* homologs of casein kinase I and II in CaM IP fractions. In humans, mutations in casein kinase I and II have been associated with abnormal circadian rythym and many forms of cancer [57,58]. In total, these results provide additional evidence linking CaM and CaMBPs to human diseases.

Mass spectrometry has also been used to reveal putative CaM interactors in human samples. For example, 297 CaM interactors were identified in various human tissues (brain, heart, spleen, thymus, and muscle) [7]. Among the identified proteins were DEAD box proteins, ribosomal proteins, and proteasomal subunits, which intriguingly, were all identified as putative CaM interactors in *Dictyostelium* (Table S1). In addition, mass spectrometry performed on proteins pulled down with affinity-tagged CaM identified 489 CaM interactors in HEK293 cells [59]. Combined, these findings show that our detection of over 500 putative CaM interactors in *Dictyostelium* is in line with studies in humans.

It is important to acknowledge that our dataset may not include all CaMBPs in *Dictyostelium*. For example, mass spectrometry did not reveal previously known CaMBPs such as the well characterized regulator of nuclear number, NumA [18]. However, the NumA-interactors, calcium-binding protein 4a (Cbp4a) (growth) and Snf12 (growth and starvation) were identified, suggesting that the interaction between NumA and CaM may have been sensitive to our IP protocol [19,32,46]. Another explanation for the exclusion of NumA and other proteins previously shown to bind CaM from our dataset (e.g., Cdk5) could be that those proteins bind to a region of CaM where the antibody binds. Therefore, our IP may have pulled down NumA-free CaM. Unfortunately, since the epitope recognized by the CaM antibody is not known, it is not possible for us to confirm this hypothesis. Technical limitations could also explain anomalies. For example, we found that we needed to extensively wash IP fractions to ensure a strong detection of CaM by LC-MS/MS. As a result, our list of putative CaMBPs may exclude weak interactors that were removed during washing. Our list may also exclude those proteins that are weakly expressed during growth and starvation.

A limitation of this study was the inability to assess whether the interactions with CaM were calcium-dependent or calcium-independent, as is done with the CaMBOT [27]. However, it is important to note that the culture medium, starvation buffer, and lysis buffer used in this study did not contain free calcium. Therefore, we speculate that putative interactors identified in CaM IP fractions were likely bound to CaM prior to cell lysis. One major benefit of our analysis was that we revealed interactions that occur *in vivo* under standard culturing conditions. This may explain why some CaMBPs that were previously identified in *in vitro* methods were not detected in our analysis. Finally, it is important to acknowledge that the proteins we revealed may not all bind CaM directly. Instead, they may interact with CaMBPs that were pulled down with CaM in the IP. In addition, some of the putative interactors could function as scaffolds that recruit non-CaMBPs (e.g., DwwA). However, since our analysis revealed previously verified CaMBPs (e.g., MhcA, CanA), and we extensively washed our IP fractions prior to LC-MS/MS, we are confident our dataset provides a thorough catalogue of putative CaMBPs in *Dictyostelium*. Finally, we cannot rule out the possibility that some of the proteins we identified were false positives. For that reason, we encourage any researcher using this catalogue of putative CaM interactors in *Dictyostelium* to independently validate proteins of interest. On the other hand, since we did not detect some proteins that were previously identified as CaMBPs in *Dictyostelium*, our catalogue might in fact underrepresent the number of CaM interactors in *Dictyostelium* during growth and starvation. 

In summary, this study provides the first catalogue of putative CaM interactors in *Dictyostelium*, which provides a valuable resource for those interested in studying the functions of CaM and CaMBPs in this model organism. Intriguingly, we did not observe a dramatic difference in the list of CaM interactors between growth and starvation, despite the significant shift in the life cycle stages (i.e., 374 CaM interactors were shared between growth and starvation). These findings indicate that CaM mediates events essential to both, and possibly all stages of the *Dictyostelium* life cycle. Along the same lines, some of the putative CaM interactors were identified in either growth or starved samples, but not both, suggesting that the binding of CaM to specific CaMBPs is dynamic, and that CaM may bind to different proteins at different stages of the *Dictyostelium* life cycle. Finally, GO term enrichment analyses revealed that only 7 of the 517 proteins identified during growth, and 9 of the 521 proteins identified during starvation, have the GO term “calmodulin-binding” associated with them suggesting that much remains to be learned about the diverse cellular and developmental roles of CaM and CaMBPs in *Dictyostelium*.

## 4. Materials and Methods

### 4.1. Cell Culture, Media, and Antibodies

AX3 cells were grown and maintained on SM/2 agar with *Klebsiella aerogenes* [28]. Cells were also grown axenically in HL5 medium at room temperature and 150 rpm. For all experiments, cells were harvested in the mid-log phase of growth (1−5 × 10^6^ cells/mL). Cultures were supplemented with 100 μg/mL ampicillin and 300 μg/mL streptomycin sulfate to prevent bacterial growth. HL5 was purchased from Formedium (Hunstanton, Norfolk, UK). KK2 buffer was composed of 0.7 g/L K_2_HPO_4_ and 2.2 g/L KH_2_PO_4_, pH 6.5. Mouse monoclonal anti-CaM (clone 6D4, C3545) was purchased from Sigma Aldrich Canada (Oakville, ON, Canada). Mouse monoclonal anti-MhcA (56-396-5) and mouse monoclonal anti-VatC (224-256-2) were purchased from the Developmental Studies Hybridoma Bank (University of Iowa, Iowa City, IA, USA) [60,61]. Rabbit polyclonal anti-Snf12 was generously provided as a gift by Dr. Danton O’Day (University of Toronto, Toronto, ON, Canada) [32]. Rabbit polyclonal anti-CtnA was generously provided as a gift by Dr. Richard Gomer (Texas A&M University, College Station, TX, USA) [62]. Goat anti-rabbit and horse anti-mouse IgG linked to HRP were purchased from New England Biolabs Canada (Whitby, ON, Canada).

### 4.2. Immunoprecipitation

Growth-phase cells were deposited into tissue culture dishes and grown overnight in HL5 to confluency. Cells were washed two times with KK2 buffer and then lysed with NP40 lysis buffer (50 mM Tris HCl, 150 mM NaCl, 0.1% NP40, pH 8.0) supplemented with a protease inhibitor tablet (Fisher Scientific Company, Ottawa, ON, Canada). Cells were also starved for 6 h in KK2 buffer prior to being lysed with NP40 lysis buffer. CaM was immunoprecipitated from whole cell lysates using SureBeads Protein G Magnetic Beads according to the manufacturer’s instructions (Bio-Rad Laboratories Limited, Mississauga, ON, Canada). Briefly, lysate (2 mg) was incubated with anti-CaM (10 µL) overnight at 4 °C on a tube rotator (head-over-tail rotation). The following day, the antibody/lysate solution was incubated with 50 μL of SureBeads pre-washed with NP40 lysis buffer (1 mL total volume) for 4 h at 4 °C on a tube rotator (head-over-tail rotation). After 4 h, a magnetic rack was used to separate the SureBeads from the supernatant (i.e., protein-depleted fraction). SureBeads were washed three times with 1 mL of NP40 lysis buffer and then once with 1 mL of buffer containing 50 mM Tris, 150 mM NaCl, pH 8.0. Isolated SureBeads were stored at −80 °C for future use.

### 4.3. SDS-PAGE and Western Blotting

CaM IP fractions were isolated by resuspending SureBeads in Laemmli sample buffer [63]. Samples were then heated at 95 °C for 5 min, after which time the supernatants were removed. Protein quantification was performed using a Qubit 2.0 Fluorometer (Fisher Scientific Company, Ottawa, ON, Canada). SDS-PAGE and western blotting for CaM was performed using a method described elsewhere [29]. SDS-PAGE and western blotting for all other proteins was performed using standard methods (2-h incubation at room temperature for primary and secondary antibodies in 5% milk/TBST). A 1:1000 dilution was used for all primary antibodies and a 1:2000 dilution for secondary antibodies. Immunoblots were digitally scanned using the ChemiDoc MP Imaging System (Bio-Rad Laboratories Limited, Mississauga, ON, Canada).

### 4.4. Mass Spectrometry and Bioinformatic Analyses

CaM IP fractions were submitted for analysis to the Mass Spectrometry Facility of the SPARC BioCentre at the Hospital for Sick Children (Toronto, ON, Canada). Briefly, samples (i.e., washed and pelleted SureBeads) were reduced with 10 mM DTT for 1 h at 60 °C, alkylated with 20 mM iodoacetamide for 45 min in the dark, and digested with trypsin (2 µg per sample) overnight at 37 °C. Samples were then lyophilized using a Speedvac vacuum concentrator, desalted using a C18 ziptip, and resuspended in 0.1% formic acid. Mass spectrometry (LC-MS/MS) was performed using the EASY-nLC 1200 nano-LC system and a Thermo Scientific Q Exactive HF-X mass spectrometer (Fisher Scientific Company, Ottawa, ON, Canada). Protein identification and quantification were performed using Proteome Discoverer (MS Amanda and Sequest HT) and Scaffold (X! Tandem), which were setup to search the Uniprot_UP000002195_*Dictyostelium*_discoideum_15092020 database according to the following parameters: parent mass error tolerance, 10 ppm; fragment mass error tolerance, 0.02 Da; enzyme, trypsin; max missed cleavages, 3; fixed modifications, carbamidomethylation (C +57.02); variable modifications, oxidation (M +15.99), deamidation (N,Q +0.98), acetylation (peptide N-term +42.01). Scaffold version 4.11.1 (Proteome Software Inc., Portland, OR, USA) was used to validate MS/MS based peptide and protein identifications. Peptide and protein identifications were accepted if they could be established at greater than 95% probability. Protein identifications were further accepted if they contained at least 2 identified peptides and were present in at least 2 of the 3 biological replicates analyzed for each treatment (growth and 6-h starvation). GO term enrichment analyses were performed using LAGO (A Logically Accelerated GO Term Finder) [64]. A *p*-value cut-off of 0.05 and the Bonferroni correction were applied for all GO term enrichment analyses. Putative CaM interactors were also analyzed using STRING database version 11, which generates a network of predicted associations for a particular group of proteins [36].

## Figures and Tables

**Table 1:** List of proteins revealed in calmodulin (CaM) immunoprecipitation (IP) fractions with the gene ontology (GO) term identifier “calmodulin-binding”.

Gene	Protein Name	Uniprot ID	dictyBase Gene ID	dictyBase Protein ID	Growth	Starvation
*canA*	Serine/threonine-protein phosphatase 2B catalytic subunit	Q7YSW8	DDB_G0276883	DDB0185021	No	Yes
*dwwA*	WW domain-containing protein A	Q54T86	DDB_G0281827	DDB0216188	Yes	Yes
*H1*	Histone H1	P54671	DDB_G0285319	DDB0191459	Yes	Yes
*mhcA*	Myosin-2 heavy chain	P08799	DDB_G0286355	DDB0191444	Yes	Yes
*myoA*	Myosin IA heavy chain	P22467	DDB_G0280039	DDB0215392	Yes	Yes
*myoE*	Myosin IE heavy chain	Q03479	DDB_G0288679	DDB0216200	Yes	Yes
*myoJ*	Myosin-J heavy chain	P54697	DDB_G0272112	DDB0185050	Yes	Yes
*rgaA*	Ras GTPase-activating-like protein rgaA	Q54K32	DDB_G0287585	DDB0191437	No	Yes
*rpl19*	60S ribosomal protein L19	P14329	DDB_G0281565	DDB0214854	Yes	Yes
gxcII *	DH domain-containing protein	Q54XW8	DDB_G0278703	DDB0233473	Yes	Yes
gxcJJ *	Rac guanine nucleotide exchange factor JJ	Q553D3	DDB_G0275679	DDB0233356	No	Yes

* Proteins that do not have the GO term identifier “calmodulin-binding” but they do have the gene descriptor “calmodulin-binding” in dictyBase.

**Table 2:** List of proteins revealed in calmodulin (CaM) immunoprecipitation (IP) fractions with identified CaM-binding domains (CaMBDs).

**CaMBPs with Canonical CaMBDs** [6]						
**Gene**	**Protein Name**	**Uniprot ID**	**dictyBase Gene ID**	**dictyBase Protein ID**	**Growth**	**Starvation**
*canA*	Serine/threonine-protein phosphatase 2B catalytic subunit	Q7YSW8	DDB_G0276883	DDB0185021	No	Yes
*mhkB*	Myosin heavy chain kinase B	P90648	DDB_G0289115	DDB0191333	No	Yes
*rgaA*	Ras GTPase-activating-like protein rgaA	Q54K32	DDB_G0287585	DDB0191437	No	Yes
*rpl19*	60S ribosomal protein L19	P14329	DDB_G0281565	DDB0214854	Yes	Yes
**CaMBPs with Non-Canonical CaMBDs** [6]						
**Gene**	**Protein Name**	**Uniprot ID**	**dictyBase Gene ID**	**dictyBase Protein ID**	**Growth**	**Starvation**
*vatM*	Vacuolar proton translocating ATPase 100 kDa subunit	Q54E04	DDB_G0291858	DDB0216215	Yes	No
**Full IQ Motifs** [6]						
**Gene**	**Protein Name**	**Uniprot ID**	**dictyBase Gene ID**	**dictyBase Protein ID**	**Growth**	**Starvation**
*mhcA*	Myosin-2 heavy chain	P08799	DDB_G0286355	DDB0191444	Yes	Yes
*myoE*	Myosin IE heavy chain	Q03479	DDB_G0288679	DDB0216200	Yes	Yes
*myoJ*	Myosin-J heavy chain	P54697	DDB_G0272112	DDB0185050	Yes	Yes
**IQ-Like Motifs** [6]						
**Gene**	**Protein Name**	**Uniprot ID**	**dictyBase Gene ID**	**dictyBase Protein ID**	**Growth**	**Starvation**
*dwwA*	WW domain-containing protein A	Q54T86	DDB_G0281827	DDB0216188	Yes	Yes
*myoA*	Myosin IA heavy chain	P22467	DDB_G0280039	DDB0215392	Yes	Yes
*myoB*	Myosin IB heavy chain	P34092	DDB_G0289117	DDB0191351	Yes	Yes
*myoC*	Myosin IC heavy chain	P42522	DDB_G0276617	DDB0215355	Yes	No
*myoI*	Myosin-I heavy chain	Q9U1M8	DDB_G0274455	DDB0185049	Yes	Yes
**Non-IQ Motifs** [6]						
**Gene**	**Protein Name**	**Uniprot ID**	**dictyBase Gene ID**	**dictyBase Protein ID**	**Growth**	**Starvation**
*myoD*	Myosin ID heavy chain	P34109	DDB_G0275447	DDB0191347	Yes	Yes

**Table 3:** Gene ontology (GO) term enrichment analysis summary (biological process) for proteins identified in calmodulin (CaM) immunoprecipitation (IP) fractions. Displaying only those results with *p* < 0.05.

GOID	Term	Growth (# of Proteins)	Starved (# of Proteins)
GO:0008152	metabolic process	337	318
GO:0010467	gene expression	231	229
GO:0009058	biosynthetic process	217	203
GO:0006412	translation	143	141
GO:0050896	response to stimulus	98	109
GO:0042254	ribosome biogenesis	86	70
GO:0006396	RNA processing	62	62
GO:0042221	response to chemical	53	59
GO:0007010	cytoskeleton organization	52	61
GO:0006950 *	response to stress	0	56
GO:0007049 *	cell cycle	0	36
GO:0006897	endocytosis	29	31
GO:0006909	phagocytosis	28	27
GO:0042255	ribosome assembly	27	23
GO:0009617	response to bacterium	22	27
GO:0000910	cytokinesis	18	22
GO:0043327 *	chemotaxis to cAMP	0	16
GO:0010038	response to metal ion	14	15
GO:0140053	mitochondrial gene expression	10	9
GO:0099518 *	vesicle cytoskeletal trafficking	9	0
GO:0006119 *	oxidative phosphorylation	9	0
GO:0030050	vesicle transport along actin filament	8	6
GO:0042026 *	protein refolding	0	6

* GOIDs that were enriched in either growth or starved samples, not both.

**Table 4:** Gene ontology (GO) term enrichment analysis summary (molecular function) for proteins identified in calmodulin (CaM) immunoprecipitation (IP) fractions. Displaying only those results with *p* < 0.05.

GOID	Term	Growth (# of Proteins)	Starved (# of Proteins)
GO:0005488	binding	345	354
GO:0003824 *	catalytic activity	202	0
GO:0003676	nucleic acid binding	176	188
GO:0043167	ion binding	168	173
GO:0003723	RNA binding	138	149
GO:0043168	anion binding	127	117
GO:0097367	carbohydrate derivative binding	122	107
GO:0003735	structural constituent of ribosome	115	109
GO:0016787 *	hydrolase activity	101	0
GO:0005524	ATP binding	88	81
GO:0016462	pyrophosphatase activity	73	65
GO:0016818	hydrolase activity, acting on acid anhydrides, in phosphorus-containing anhydrides	73	65
GO:0008092	cytoskeletal protein binding	35	43
GO:0016887	ATPase activity	34	34
GO:0005525 *	GTP binding	28	0
GO:0003924 *	GTPase activity	22	0
GO:0005509 *	calcium ion binding	0	21
GO:0004386	helicase activity	20	19
GO:0003743	translation initiation factor activity	17	21
GO:0016779 *	nucleotidyltransferase activity	16	0
GO:0003899 *	DNA-directed 5’–3’ RNA polymerase activity	13	0
GO:0034062 *	5’–3’ RNA polymerase activity	13	0
GO:0097747 *	RNA polymerase activity	13	0
GO:0003682 *	chromatin binding	0	11
GO:0046983	protein dimerization activity	11	10
GO:0003774	motor activity	9	8
GO:0043022 *	ribosome binding	8	0
GO:0005516	calmodulin binding	7	9

* GOIDs that were enriched in either growth or starved samples, not both.

**Table 5:** Gene ontology (GO) term enrichment analysis summary (cellular component) for proteins identified in calmodulin (CaM) immunoprecipitation (IP) fractions. Displaying only those results with *p* < 0.05.

GOID	Term	Growth (# of Proteins)	Starved (# of Proteins)
GO:0005737	cytoplasm	326	325
GO:0005634	nucleus	147	155
GO:0005829	cytosol	123	119
GO:0005840	ribosome	122	115
GO:0031982	vesicle	104	105
GO:0005739	mitochondrion	90	64
GO:0030139	endocytic vesicle	89	89
GO:0045335	phagocytic vesicle	88	88
GO:0005576	extracellular region	69	70
GO:0005730	nucleolus	67	55
GO:0005856	cytoskeleton	47	53
GO:0071944 *	cell periphery	0	50
GO:0031090 *	organelle membrane	43	0
GO:0005759	mitochondrial matrix	40	31
GO:0005761	mitochondrial ribosome	29	23
GO:0005938	cell cortex	29	36
GO:0005694	chromosome	26	28
GO:0031966	mitochondrial membrane	23	23
GO:0005811	lipid droplet	22	14
GO:0005743 *	mitochondrial inner membrane	22	0
GO:0000785	chromatin	20	18
GO:0031143	pseudopodium	15	21
GO:0005746 *	mitochondrial respiratory chain	10	0
GO:0030666	endocytic vesicle membrane	10	10
GO:0005905	clathrin-coated pit	7	10
GO:0012506 *	vesicle membrane	0	15
GO:0070938 *	contractile ring	0	4

* GOIDs that were enriched in either growth or starved samples, not both.

## Supplementary Materials

Supplementary materials can be found at https://www.mdpi.com/1422-0067/22/4/1722/s1.

## Abbreviations

CaM	calmodulin
CaMBP	CaM-binding protein
CaMBD	CaM-binding domain
CaMBOT	CaM-binding overlay technique
cAMP	cyclic adenosine monophosphate
CanA	calcineurin A
CtnA	countin
CTSD	cathepsin D
DwwA	WW domain-containing protein A
GO	Gene ontology
GxcJJ	guanine exchange factor for rac JJ
H1	histone H1
LC-MS/MS	liquid chromatography-mass spectrometry/mass spectrometry
HEXA	hexosaminidase A
IP	immunoprecipitation
MhcA	myosin II heavy chain
NagA	N-acetylglucosaminidase A
NCL	neuronal ceroid lipofuscinosis
NumA	nucleomorphin A
RgaA	Ras GTPase-activating-like protein
Snf12	SWI/SNF protein 12
VatC	V-ATPase catalytic subunit
